# Effects of switching from sacubitril/valsartan to valsartan alone on plasma levels of natriuretic peptides and myocardial remodeling in heart failure with reduced ejection fraction

**DOI:** 10.1186/s12872-023-03077-2

**Published:** 2023-01-21

**Authors:** Akihiro Nakamura, Yuta Kagaya, Hiroki Saito, Masanori Kanazawa, Masanobu Miura, Masateru Kondo, Kenjiro Sato, Hideaki Endo

**Affiliations:** Department of Cardiology, Iwate Prefectural Central Hospital, 1-4-1 Ueda, Morioka, Iwate 020-0066 Japan

**Keywords:** Angiotensin-receptor blocker, Angiotensin receptor-neprilysin inhibitor, Myocardial remodeling, Natriuretic peptide, Wall stress

## Abstract

**Background:**

We examined the effect of switching from angiotensin receptor-neprilysin inhibitor (ARNI) to angiotensin-receptor blocker (ARB) on plasma levels of natriuretic peptides and myocardial remodeling.

**Methods:**

This is a prospective study that included 11 patients with heart failure (HF) treated with ARNI. The patients were divided into two groups: 5 patients who continued treatment with sacubitril/valsartan 194/206 mg/day (ARNI-continue group) and 6 patients who were switched to valsartan 160 mg/day (ARB-switch group). The primary endpoint was percent change (%Change) in plasma A-, B-, and N-terminal pro-B-type natriuretic peptide (ANP, BNP, and NT-proBNP) levels from the baseline to week 24. The secondary endpoint was the change in echocardiographic parameters related to myocardial remodeling from the baseline to week 24.

**Results:**

ANP levels in the ARB-switch group significantly decreased (from 1155.7 ± 592.6 pg/mL to 231.6 ± 233.8 pg/mL, *p* = 0.035), whereas those in the ARNI-continue group were not significant (*p* = 0.180). The %Change of decrease in ANP levels was significantly greater in the ARB-switch group than the ARNI-continue group (− 76.9% vs. −9.1%, *p* = 0.009). BNP levels were not significantly different between the baseline and week 24 in both groups. NT-proBNP levels in the ARB-switch group increased from 1185.3 ± 835.6 pg/mL to 1515.2 ± 1213.5 pg/mL, although the changes were not statistically significant (*p* = 0.345). The %Change of increase in NT-proBNP levels was significantly greater in the ARB-switch group than the ARNI-continue group (57.9% vs. 17.3%, *p* = 0.016). In the ARB-switch group, there was a significant increase in left ventricular (LV) end-systolic volume (from 41.3 ± 24.1 mL/m^2^ to 71.4 ± 8.8 mL/m^2^, *p* = 0.043) and LV peak-systolic wall stress (from 187.0 ± 42.7 × 10^3^ dynes/cm^2^ to 279.7 ± 34.1 × 10^3^ dynes/cm^2^, *p* = 0.012) from the baseline to week 24 and a trend toward a decrease in LV ejection fraction (*p* = 0.080). In the ARNI-continue group, no differences in echocardiographic parameters were observed from the baseline to week 24.

**Conclusion:**

Switching from ARNI to ARB may worsen HF due to returning to myocardial remodeling induced by a sustained decline in ANP levels.

## Background

The increasing number of patients with heart failure (HF) is an international economic and health-related issue [1, 2]. Currently, the estimated number of patients with HF has been reported to be approximately 1,000,000 in Japan, which is assumed to reach 1,300,000 by 2035 with the rapid aging of society [2, 3]. Recently, there have been significant advances in the development of drugs for HF treatment. However, it is still necessary to search for new pharmacological targets and innovate new drugs for patients with HF.

Sacubitril/valsartan is a first-in-class angiotensin receptor-neprilysin inhibitor (ARNI) and one of the promising novel drugs for advanced management of HF. In the clinical phase III trial, ARNI demonstrated a lower rate of hospitalization for HF or death from cardiovascular causes than enalapril, an angiotensin-converting enzyme inhibitor (ACEI), in patients with HF with reduced ejection fraction (HFrEF) (≤ 40%) [4]. However, a more frequent symptomatic hypotension has been known in patients with ARNI than in those with ACEI [4, 5]. In case of difficulty in continuing administration of ARNI due to any side effects such as hypotension or financial shortage, switching from ARNI to angiotensin- receptor blocker (ARB) alone would be one of the therapeutic options.

In the literature, a large amount of data has been published on the efficacy and safety of switching from ACEI or ARB to ARNI [6]. However, data on switching from ARNI to ACEI or ARB are not available. Patients with HF are commonly founded to have variations in myocardial geometry and left ventricular (LV) function, which were evaluated by a standard echocardiographic examination. These echocardiographic parameters have associated with circulating cardiac biomarkers such as plasma A-, B-, and N-terminal pro-B-type natriuretic peptide (ANP, BNP, and NT-proBNP) which are commonly measured to diagnose and treat HF [7, 8].

Thus, this small prospective study aimed to examine the changes in plasma natriuretic peptide levels, echocardiographic parameters related to myocardial remodeling, and LV wall stress (WS) from the baseline to week 24 in patients who were switched from ARNI to ARB alone compared with patients who continued treatment with ARNI.

## Methods

### Patients and study design

**Fig. 1:**
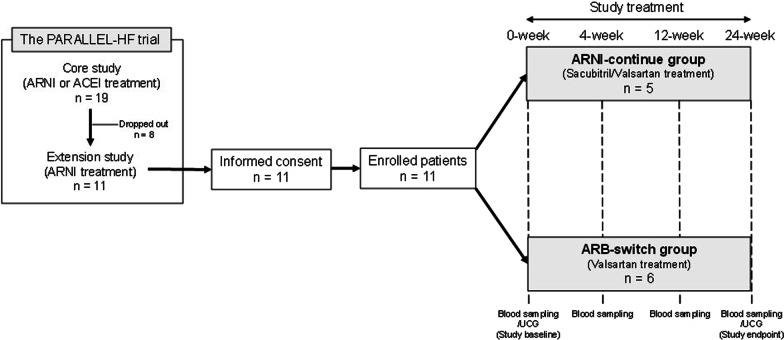
The flowchart of this study. PARALLEL-HF, prospective comparison of ARNI with ACEI to determine the novel beneficial treatment value in Japanese heart failure patients. *ARNI* angiotensin receptor-neprilysin inhibitor; *ACEI* angiotensin-converting enzyme inhibitor; *UCG* ultrasound cardiography

A total of 19 patients with chronic HF (New York Heart Association class II–IV; LV ejection fraction (LVEF) ≤ 35%) in our hospital between November 2015 and December 2016 participated in the PARALLEL-HF (prospective comparison of ARNI with ACEI to determine the novel beneficial treatment value in Japanese HF patients) trial which was conducted to confirm efficacy and safety of ARNI (sacubitril/valsartan) versus ACEI (enalapril) in Japanese patients with HFrEF [4]. This trial had two study parts: the first was a core study (double-blind, ARNI or ACEI treatment period), and the second was an extension study (open-label, active drug ARNI treatment period). Out of the 19 patients, eight dropped out during the core study period. In the next extension study, 11 patients were administered ARNI (sacubitril/valsartan 194/206 mg/day) until December 2020 to January 2021 after the core study. Before the end of the study, they were informed about the core study outcomes, which revealed an advantage of ARNI over ACEI regarding the prevention of cardiovascular death or rehospitalization for worsening HF in patients with reduced EF [4]. Despite the recommendation to continue the ARNI treatment, six of 11 patients desired to change the treatment from ARNI to ARB because of the medical costs or the two-week visit after the study. The remaining five patients wanted to continue the ARNI treatment. After completing the core study between December 2020 and January 2021, two groups were enrolled in this study: five patients who requested the continuous treatment with ARNI (sacubitril/valsartan 194/206 mg/day) (ARNI-continue group) and six patients who requested the treatment with ARB (valsartan 160 mg/day) (ARB-switch group). The follow-up visits were conducted at 4, 12, and 24 weeks (Fig. 1).

The primary endpoint was percent change (%Change) in plasma ANP, BNP, and NT-proBNP levels from the baseline to week 24. The secondary endpoint was the change in echocardiographic parameters from the baseline to week 24. %Change was calculated as follows: ([value at the 24-week treatment  − value at the baseline] / value at the baseline) × 100.

This prospective, open-label, non-randomized, single-center study was approved by the ethics committee of the Iwate Prefectural Central Hospital, Iwate, Japan (approval no. 1903) and conducted in accordance with the principles of the Declaration of Helsinki. Written informed consent was obtained from all patients. This study was registered with the University Hospital Medical Information Network Clinical Trials Registry (UMIN000048602, 05/08/2022).

### Laboratory examinations

Blood samples at rest were collected from the antecubital vein at the baseline and at weeks 4, 12, and 24. Plasma levels of ANP and BNP (in pg/mL) were measured at the baseline and at weeks 4, 12, and 24, and those of NT-proBNP (in pg/mL) were measured at the baseline and at week 24. The baseline data were measured within one month of the enrollment. Plasma levels of ANP were measured using a commercially available chemiluminescent enzyme immunoassay system (SRL, Tokyo, Japan), and those of BNP and NT-proBNP were measured using an ARCHITECT BNP-JP chemiluminescence immunoassay (Abbott Laboratories, Abbott Park, IL, USA) and an Elecsys proBNP II electrochemiluminescence immunoassay (Roche Diagnostics, Basel, Switzerland), respectively, in the clinical laboratory of our hospital.

### Echocardiographic examinations

Echocardiography was performed in all patients at the baseline and at week 24 using a commercially available ultrasound system (Vivid E90, General Electric Healthcare, Horten, Norway). At the time of echocardiography, blood pressure (BP) measurements were conducted twice on the non-dominant arm after at least 10 min of rest using Omron HCR-7201 device (Omron, Kyoto, Japan), and the results were averaged. Standard images based on the apical 4-chamber, 2-chamber, and parasternal long- and short-axis views were recorded and analyzed by one experienced sonographer (M.A.) and confirmed by a second echocardiography expert (K.F.) who were blinded to the study protocol, according to the criteria of the American Society of Echocardiography and the European Association of Cardiovascular Imaging [9]. LV end-diastolic volume (LVEDV, in mL) and LV end-systolic volume (LVESV, in mL) were measured from the apical 4- and 2-chamber views using the modified Simpson’s biplane method and normalized by body surface area (LVEDV index, in ml/m^2^; LVESV index, in ml/m^2^, respectively). LVEF (in %) was calculated using the cube method as follows: LVEF = ([LVEDV − LVESV] / LVEDV) × 100 [9]. Left atrial volume (LAV, in mL) was measured from standard apical 4-chamber views at end-systole before mitral valve opening using the modified Simpson’s method [10] and normalized by body surface area (LAV index, in ml/m^2^). With two-dimensional imaging taken from parasternal long-axis view, LV mass (LVM, in g) was calculated using the Devereux formula with modification as follows [11, 12]: LVM = 0.8 × 1.04 × [(LVDd + IVSTd + PWTd)^3^ − LVDd^3^] + 0.6, where LVDd is the LV dimension (LVD) at end-diastole, IVSTd is the interventricular septal thickness (IVST) at end-diastole, and PWTd is the posterior wall thickness (PWT) at end-diastole. LV mass index (in g/m^2^) was also determined as the LVM (in g) divided by body surface area (in m^2^). LV meridional WS was calculated using the validated formula as follows [13, 14]: WS = (0.334 × systolic BP × LVD) / {PWT × [1 + (PWT/LVD)]}. For end-systolic WS (ESWS, in 10^3^ dynes/cm^2^), LVD is LVDs and PWT is PWTs. For peak-systolic WS (PSWS, in 10^3^ dynes/cm^2^), LVD is LVDd and PWT is PWTd.

### Statistical analysis

Continuous variables were expressed as mean ± standard deviation, and categorical variables were expressed as numbers and percentages. The between-group and the within-group differences for continuous variables were assessed using the Mann–Whitney U test and the Wilcoxon signed-rank test, respectively. The categorical variables for the between-group differences were examined using Fisher’s exact test. The time-course patterns of systolic and diastolic BP, %Change in plasma ANP, and BNP levels in each group were analyzed using a repeated- measures single-factor analysis of variance (ANOVA). All statistical analyses were performed using Excel (Microsoft, Redmond, WA, USA) with add-in software Statcel4. A *p*-value < 0.05 was considered statistically significant.

## Results

### Patient enrollment and baseline characteristics

A total of 11 patients were enrolled in our study. The baseline characteristics of patients in the ARNI-continue (*n* = 5) and the ARB-switch (*n* = 6) groups are shown in Table 1. The mean age of the ARB-switch group was significantly lower than that of the ARNI-continue group (*p* = 0.028). No significant differences in sex, body mass index, body surface area, heart rate, BP, risk factors, biochemical data, and medications were observed between the two groups.

**Table 1:** Baseline characteristics of patients in the ARNI-continue and ARB switch-groups

Variables	Total (*n* = 11)	ARNI-continue group (*n* = 5)	ARB-switch group (*n* = 6)	*p*-value
Age, year	68.1 ± 10.9	76.6 ± 5.1	61.0 ± 9.3	0.028
Male gender, *n* (%)	8 (73)	3 (60)	5 (83)	0.333
Weight, kg	67.3 ± 10.1	66.7 ± 3.3	67.8 ± 13.9	0.624
Body mass index, kg/m^2^	25.1 ± 3.8	25.0 ± 2.9	25.2 ± 4.7	1.000
Body surface area, m^2^	1.73 ± 0.12	1.73 ± 0.05	1.73 ± 0.17	0.621
Heart rate, beats/min	69 ± 11	73 ± 12	66 ± 10	0.409
Heart rhythm disorder				
Atrial fibrillation, *n* (%)	3 (27)	1 (20)	2 (33)	0.576
Pacing, *n* (%)	1 (9)	1 (20)	0 (0)	0.455
Systolic BP, mmHg	123 ± 16	117 ± 18	128 ± 14	0.325
Diastolic BP, mmHg	77 ± 14	66 ± 14	83 ± 7	0.045
Risk factor				
Previous or current hypertension, *n* (%)	6 (55)	2 (40)	4 (67)	0.392
Previous or current DM, *n* (%)	5 (45)	4 (80)	1 (17)	0.067
Previous or current dyslipidemia, *n* (%)	6 (55)	3 (60)	3 (50)	0.608
Previous or current smoking, *n* (%)	6 (55)	2 (40)	4 (67)	0.392
NYHA class ≥ III, *n* (%)	3 (27)	1 (20)	2 (33)	0.576
Ischemic heart failure etiology, *n* (%)	5 (45)	3 (60)	2 (33)	0.392
Biochemical data				
Serum creatinine, mg/dL	1.1 ± 0.4	1.1 ± 0.4	1.2 ± 0.4	0.855
eGFR, mL/min/1.73 m^2^	54.7 ± 19.9	54.0 ± 25.2	55.4 ± 15.9	0.754
ANP (pg/mL)	908.4 ± 635.6	611.6 ± 474.6	1155.7 ± 592.6	0.144
BNP (pg/mL)	375.2 ± 296.7	195.4 ± 141.5	524.9 ± 311.1	0.100
NT-proBNP (pg/mL)	1003.7 ± 753.2	785.8 ± 457.9	1185.3 ± 835.6	0.584
ARNI administration period before enrollment, months	40 ± 17	29 ± 16	48 ± 13	0.062
Medications at enrollment				
β-blockers, *n* (%)	11 (100)	5 (100)	6 (100)	1.000
Loop diuretics, *n* (%)	9 (82)	4 (80)	5 (83)	0.727
Aldosterone antagonists, *n* (%)	5 (45)	2 (40)	3 (50)	0.608
Calcium sensitizers, *n* (%)	2 (18)	2 (40)	0 (0)	0.182
SGLT-2 inhibitors, *n* (%)	1 (9)	0 (0)	1 (17)	0.546
Statins, *n* (%)	7 (64)	3 (60)	4 (67)	0.500
Antiarrhythmic drugs, *n* (%)	1 (9)	0 (0)	1 (17)	0.546
Echocardiographic data				
LVEDV index, mL/m^2^	67.2 ± 29.2	62.3 ± 24.2	71.2 ± 35.0	0.806
LVESV index, mL/m^2^	39.6 ± 21.5	37.5 ± 21.2	41.3 ± 24.1	0.806
LVEF, %	43.2 ± 11.1	42.8 ± 11.4	43.6 ± 12.2	1.000
PWTd, mm	11.3 ± 1.5	11.8 ± 1.5	11.0 ± 1.6	0.451
PWTs, mm	13.8 ± 2.2	14.5 ± 2.6	13.2 ± 1.8	0.451
IVSTd, mm	10.7 ± 1.4	11.3 ± 1.5	10.2 ± 1.3	0.447
IVSTs, mm	13.1 ± 2.0	13.3 ± 2.1	13.0 ± 2.1	0.898
LVM index, g/m^2^	139.6 ± 27.7	138.7 ± 35.1	140.0 ± 24.7	0.624

Values are mean ± standard deviation or number (%)

*ARNI* angiotensin receptor-neprilysin inhibitor; *ARB* angiotensin II receptor blocker; *BP* blood pressure; *DM* diabetes mellitus; *NYHA* New York Heart Association; *eGFR* estimated glomerular filtration rate; *ANP* A-type natriuretic peptide; *BNP* B-type natriuretic peptide; *NT-proBNP* N-terminal pro-B-type natriuretic peptide; *SGLT-2* sodium-glucose cotransporter 2; *LVEDV* left ventricular end-diastolic volume; *LVESV* left ventricular end-diastolic volume; *LVEF* left ventricular ejection fraction; *LVM* left ventricular mass, *PWTd* posterior wall thickness at end-diastole; *PWTs* posterior wall thickness at end-systole; *IVSd* interventricular septal thickness at end-diastole; *IVSs* interventricular septal thickness at end-systole; *WS* wall stress; *ESWS* end-systolic wall stress; *PSWS* peak-systolic wall stress

### Changes in BP

**Fig. 2:**
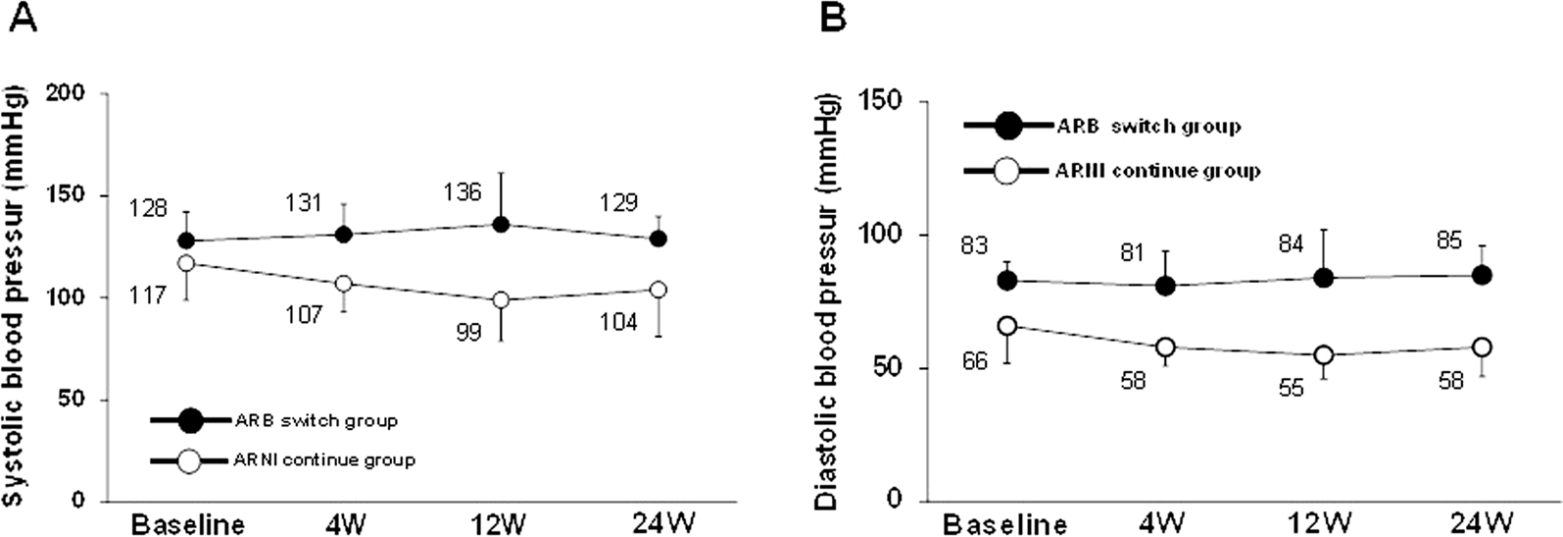
BP changes over time in the ARB-switch and ARNI-continue groups. (**A**) Systolic BP and (**B**) diastolic BP. Values are mean ± standard deviation. *ARB* angiotensin-receptor blocker; *ARNI* angiotensin receptor-neprilysin inhibitor

The changes in mean systolic and diastolic BP from the baseline to week 24 in the two groups are shown in Fig. 2. Systolic (Fig. 2 A) and diastolic BP levels (Fig. 2B) gradually increased in the ARB-switch group and decreased in the ARNI-continue group at week 12 from the baseline; however, there were no statistically significant changes in systolic and diastolic BP during overall time course in both groups (systolic and diastolic BP in the ARB-switch group, *p* = 0.871 and 0.969 by ANOVA; systolic and diastolic BP in the ARNI-continue group, *p* = 0.580 and 0.453 by ANOVA, respectively).

### Changes in plasma natriuretic peptides levels

The %Change in the plasma ANP levels during the 24-week treatment was significantly greater in the ARB-switch group than that in the ARNI-continue group (*p* = 0.009) (Table 2). Furthermore, the %Change in the plasma BNP levels during the 24-week treatment was not significantly different between the two groups (*p* = 0.175), whereas the %Change in the plasma NT-proBNP levels was significantly greater in the ARB-switch group than that in the ARNI-continue group (*p* = 0.016).

**Table 2:** %Change in natriuretic peptides from the baseline to the study endpoint after the 24-week treatment with ARNI or ARB

	ARNI-continue group (*n* = 5)		ARB-switch group (*n* = 6)		*p*-value
		%Change		%Change	(%Change)
ANP (pg/mL)					
Baseline	611.6 ± 474.6		1155.7 ± 592.6		
24 weeks	582.4 ± 511.7	− 9.1 ± 35.6	231.6 ± 233.8*	− 76.9 ± 15.9	0.009
BNP (mg/mL)					
Baseline	195.4 ± 141.5		524.9 ± 311.1		
24 weeks	235.0 ± 207.6	0.6 ± 37.0	293.7 ± 232.8	− 45.8 ± 38.2	0.175
NT-proBNP (pg/mL)					
Baseline	785.8 ± 457.9		1185.3 ± 835.6		
24 weeks	880.6 ± 619.8	17.3 ± 24.1	1515.2 ± 1213.5	57.9 ± 29.1	0.016

Values are presented as mean ± standard deviation

*ARNI* angiotensin receptor-neprilysin inhibitor; *ARB* angiotensin II receptor blocker; *ANP* A-type natriuretic peptide; *BNP* B-type natriuretic peptide; *NT-proBNP* N-terminal pro-B-type natriuretic peptide; *%Change* percent change

*p < 0.05 versus the baseline value in the same group

**Fig. 3:**
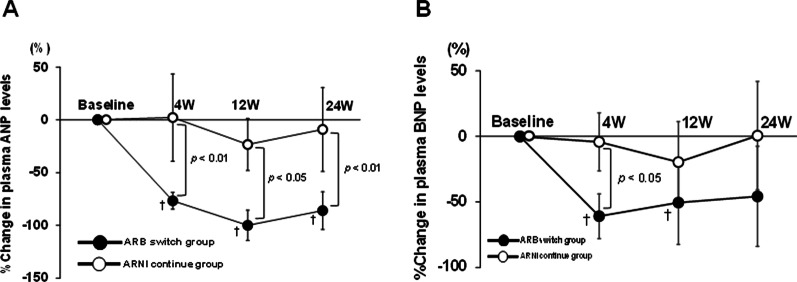
%Change in plasma levels of ANP (**A**) and BNP (**B**) in the ARB-switch and ARNI-continue groups. Values are mean ± standard deviation. ^†^*p* < 0.01 versus the baseline value. *ANP* A-type natriuretic peptide; *BNP* B-type natriuretic peptide; *ARB* angiotensin-receptor blocker; *ARNI* angiotensin receptor-neprilysin inhibitor

The changes in the plasma ANP and BNP levels over time are shown in Fig. 3. The ARNI-continue group showed no significant difference in the plasma ANP levels during 24 weeks, whereas the ARB-switch group showed a remarkable decrease in the plasma ANP levels from the baseline to week 4 (from 1155.7 ± 649.1 pg/mL to 206 ± 103.3 pg/mL, *p* = 0.028) and reduced ANP levels at weeks 12 and 24 (239.3 ± 200.2 pg/mL at week 12 and 231.6 ± 233.8 pg/mL at week 24 vs. 1155.7 ± 592.6 pg/mL at the baseline, *p* = 0.023 and *p* = 0.043, respectively) (Fig. 3 A). The ARNI-continue group showed no significant difference in the plasma BNP levels during 24 weeks, whereas the ARB-switch group showed a significant decrease in the plasma BNP levels from the baseline to weeks 4 and 12 (from 524.9 ± 311.1 pg/mL at the baseline to 183.8 ± 95.2 pg/mL at week 4, *p* = 0.028; 270.7 ± 206.8 pg/mL at week 12, *p* = 0.046) (Fig. 3B). Furthermore, the ARB-switch group showed no significant difference in the plasma BNP levels between the baseline and week 24 (*p* = 0.080) (Table 2).

### Changes in echocardiographic parameters

The changes in echocardiographic parameters related to myocardial remodeling from the baseline to week 24 in the two groups are shown in Table 3. The ARNI-continue group showed no significant differences in LVEDV, LVESV, LVEF, LAV, and LVM values between the baseline and week 24, whereas the ARB-switch group showed a significant increase in the LVESV value (*p* = 0.043) and a trend toward a decrease in the LVEF value (*p* = 0.080) from the baseline to week 24. Furthermore, the ARB-switch group showed no significant difference in LVEDV, LAV, and LVM values between the baseline and week 24.

**Table 3:** Change in echocardiographic parameters from the baseline to the study endpoint after the 24-week treatment with ARNI or ARB

	ARNI-continue group (*n* = 5)		*p*-value	ARB-switch group (*n* = 6)		*p*-value
	Baseline	24 weeks		Baseline	24 weeks	
LVEDV index, mL/m^2^	62.3 ± 24.2	51.1 ± 19.1	0.465	71.2 ± 35.0	103.5 ± 6.8	0.138
LVESV index, mL/m^2^	37.5 ± 21.2	27.4 ± 14.1	0.465	41.3 ± 24.1	71.4 ± 8.8	0.043
LVEF, %	42.8 ± 11.4	44.8 ± 17.2	0.715	43.6 ± 12.2	30.6 ± 9.1	0.080
LAV index, mL/m^2^	42.3 ± 8.7	38.0 ± 9.7	0.109	46.2 ± 12.5	55.8 ± 11.9	0.225
LVM index, g/m^2^	138.7 ± 35.1	128.6 ± 8.2	0.715	140.0 ± 24.7	143.1 ± 11.7	0.686

Values are presented as mean ± standard deviation

*ARNI* angiotensin receptor-neprilysin inhibitor; *ARB* angiotensin II receptor blocker; *LVEDV* left ventricular end-diastolic volume; *LVESV* left ventricular end-systolic volume; *LVEF* left ventricular ejection fraction; *LAV* left atrial volume; *LVM* left ventricular mass

### Changes in LV systolic WS

**Fig. 4:**
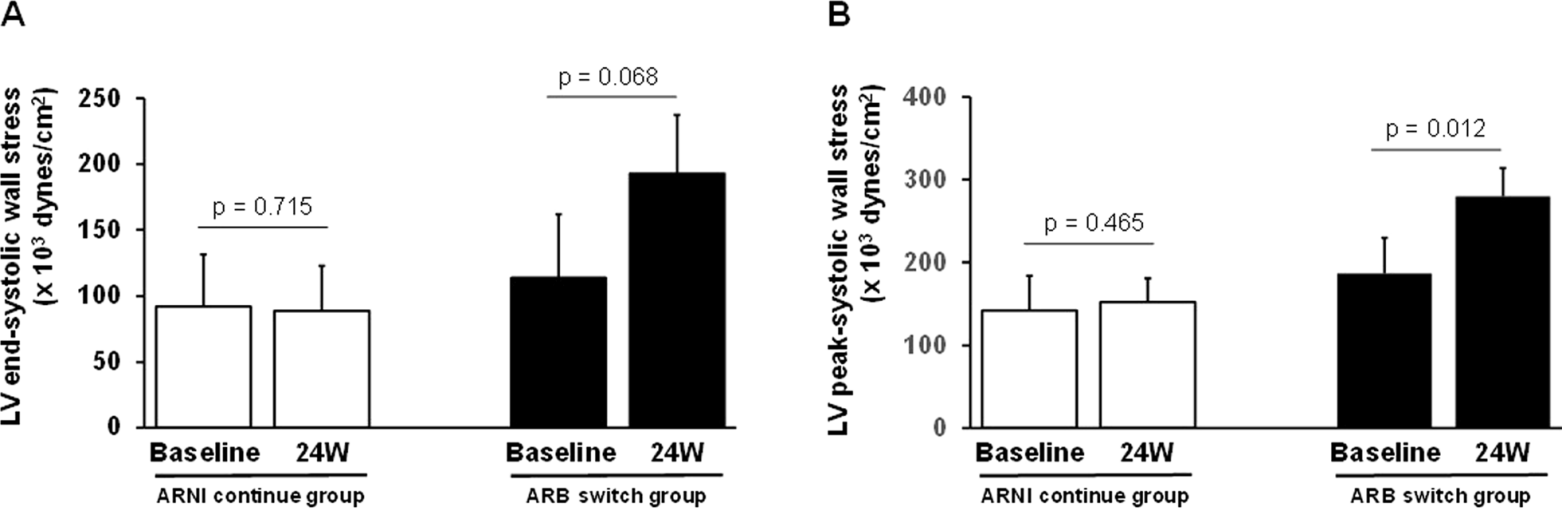
Comparison of LV WS between the baseline and week 24 in the ARB-switch and ARNI-continue groups. (**A**) ESWS and (**B**) PSWS. Values are mean ± standard deviation. *ARNI* angiotensin receptor-neprilysin inhibitor; *ARB* angiotensin-receptor blocker

The LV ESWS and PSWS at the baseline and week 24 in the two groups are shown in Fig. 4. In the ARNI-continue group, the ESWS value was not significantly different between the baseline and week 24 (92.0 ± 39.3 × 10^3^ dynes/cm^2^ at the baseline vs. 89.0 ± 34.0 × 10^3^ dynes/cm^2^ at week 24, *p* = 0.715), whereas in the ARB-group, it was greater at week 24 than at the baseline (192.8 ± 44.5 × 10^3^ dynes/cm^2^ at week 24 vs. 113.9 ± 48.0 × 10^3^ dynes/cm^2^ at the baseline); however, the difference was not statistically significant (*p* = 0.068) (Fig. 4 A). In the ARNI-continue group, the PSWS value was not significantly different between the baseline and week 24 (142.1 ± 42.2 × 10^3^ dynes/cm^2^ at the baseline vs. 152.4 ± 28.9 × 10^3^ dynes/cm^2^ at week 24, *p* = 0.465), whereas in the ARB-group, it was significantly greater at week 24 than at the baseline (279.7 ± 34.1 × 10^3^ dynes/cm^2^ at week 24 vs. 187.0 ± 42.7 × 10^3^ dynes/cm^2^ at the baseline, *p* = 0.012) (Fig. 4B). The %Changes in ESWS and PSWS during the 24-week treatment were not significantly different (ESWS, *p* = 0.149; PSWS, *p* = 0.086).

The LVM index was not significantly different between the baseline and week 24 in both groups (ARNI-continue group, 161.4 ± 38.2 g/m^2^ at the baseline vs. 167.5 ± 59.9 g/m^2^ at week 24, *p* = 0.700; ARB-switch group, 128.1 ± 7.6 g/m^2^ at the baseline vs. 129.2 ± 5.9 g/m^2^ at week 24, *p* = 0.735).

### Cardiovascular events

The ARNI or ARB treatment was not changed or discontinued in all patients during the study period. No patients were given additional medications for HF such as sodium-glucose cotransporter-2 inhibitors. Additionally, unplanned hospitalization due to worsening HF or other cardiovascular events was not observed in both groups.

## Discussion

This small prospective, non-randomized study demonstrated that plasma ANP and BNP levels continuously decreased in patients with HF with switching from sacubitril/valsartan of 194/206 mg/day to valsartan of 160 mg/day. Furthermore, the increased NT-proBNP levels suggest an increase in cardiac afterload, thus decreasing LV systolic performance by 24 weeks at the latest.

The ARNI has become the mainstay of treatment in patients with HFrEF after the PARADIGM-HF trial demonstrating a decreased risk of cardiovascular death or HF hospitalization compared to enalapril with those patients [4, 15, 16]. By and large, the majority of those patients benefit from the treatment with ARNI. However, some patients have the potential to suffer from adverse effects such as hypotension, hyperkalemia, renal failure, and angioedema [4, 17, 18]. The PARADIGM-HF trial demonstrated a higher frequency of hypotension and nonserious angioedema in patients with ARNI than in those with ACEI. However, the proportion of patients who discontinued medication did not differ between the ARNI and ACEI groups [4]. Because these side effects are not negligible, especially hypotension in patients under severe cardiac or renal conditions, it is meaningful to examine whether switching from ARNI to ARB or ACEI alone could be an alternative option. From an ethical point of view, it is not easy to conduct such studies in stable patients with HF during the ARNI treatment. Thus, our study enrolled the patients who had to decide whether to continue the ARNI treatment for a fee after the PARADIGM-HF trial.

Natriuretic peptides are partially degraded by neprilysin and cleared from circulation. Affinity for neprilysin is higher in ANP than in BNP, resulting in higher plasma levels of ANP than BNP when inhibited [19–21]. Nougué et al. reported an approximately 4-fold increase in plasma ANP levels, while there was no change in plasma BNP levels and its activity in 73 patients with HFrEF who were switched from ARB or ACEI to ARNI [19]. Ibrahim et al. observed the consistently and substantially increased change in ANP levels and the inconsistent change in BNP levels in 23 stable patients with HFrEF who were initiated and titrated on ARNI treatment [19]. In our study, the ARB-switch group showed a larger and more sustained decrease (an approximately 5-fold decrease) in the plasma ANP levels and a modest decrease (an approximately 3-fold decrease) in plasma BNP levels after the release of neprilysin inhibition. These results are compatible with the above previous studies. To our knowledge, this is the first report to demonstrate the changes in plasma ANP and BNP levels after the discontinuation of the ARNI treatment, even if it was a study of a small number of cases.

Favorable pathophysiological effects such as reverse myocardial remodeling by the neprilysin inhibition in patients with HF have been reported to be potentially mediated by the increase in plasma ANP levels rather than that of plasma BNP levels [19–21]. Murphy et al. reported that a rapid and greater increase in plasma ANP levels was associated with a larger increase in LVEF and a decrease in LAV in 144 patients with HFrEF who initiated and titrated on ARNI treatment [21]. Our study also showed a decrease in LVEF and an increase in LAV index during 24 weeks in the ARB-switch group, although the differences were not statistically significant. From the viewpoint of myocardial mechanics related to the LV remodeling, the cardiac systolic load can be estimated by LV systolic WS, which is a significant determinant of LV systolic performance [22, 23]. In our study, the increase in both plasma NT-proBNP levels and LV systolic WS was observed at week 24 in the ARB-switch group. Because NT-proBNP is a biomarker reflecting cardiac WS [24], this biomarker may be an important determinant of systolic performance after the discontinuation of the ARNI treatment. Despite the increased afterload due to the decrease in ANP levels, BP remained unchanged in the ARB-switch group. This finding suggests the deterioration of LV systolic performance when switching from ARNI to ARB alone.

## Study limitations

Our study had several limitations. First, the number of patients is rather small, which may limit the significance of statistical analysis. However, the changes in plasma natriuretic peptides levels, especially ANP levels were robust and highly significant. Because these changes and the myocardial remodeling when switching from ARNI to ARB alone have not been fully evaluated, we believe our study will provide an important information on the risk of releasing neprilysin inhibition. Second, the substrates for neprilysin other than natriuretic peptides, such as endothelin, adrenomedullin, substance P, and angiotensin II [25], were not assessed. Thus, it remains unclear how much these peptides are involved in myocardial remodeling. Third, the treatment of the patients in our study was switched from ARNI to ARB. Therefore, data on patients switching from ARNI to ACEI are not available.

## Conclusion

Switching from ARNI to ARB decreased plasma ANP and BNP levels. However, it increased plasma NT-proBNP levels and reduced LV systolic performance by 24 weeks at the latest. These results showed that patients with HF should be carefully monitored to be aware of worsening HF after switching from ARNI to ARB.

## Data Availability

The datasets generated and/or analyzed during the current study are not publicly available due to privacy or ethical restrictions but are available from the corresponding author on reasonable request.
